# Upregulation of Brain's Calcium Binding Proteins in Mitragynine Dependence: A Potential Cellular Mechanism to Addiction

**DOI:** 10.7150/ijms.78861

**Published:** 2023-01-01

**Authors:** Murtadha Basheer, Zurina Hassan, Lay-Harn Gam

**Affiliations:** 1 School of pharmaceutical sciences, Universiti Sains Malaysia, Penang, Malaysia; 2 Center of Drug Research, Universiti Sains Malaysia, Penang, Malaysia

**Keywords:** Mitragynine, animal behavioral study, proteomics, calcium-related proteins

## Abstract

**Background:*** Mitragyna speciosa* Korth or Kratom is widely used traditionally for its medicinal values. The major alkaloid content of kratom leaves is mitragynine, which binds to opioid receptors to give opioid-like effects. This study aimed to analyse the brain proteome of animals that displayed addictive behaviors.

**Design and Methods:** Six groups (n=6-8) of rats made up of negative control, positive control using morphine (10 mg/kg), and treatment groups at low (1mg/kg) and high doses of mitragynine (30 mg/kg) for 1 and 4 days. The rats' behaviors were evaluated and subsequently the rats' brains were harvested for proteomic analysis that was performed by using 2D gel electrophoresis and LC/MS/MS.

**Results:** The rats developed physical dependence only on day 4 following morphine and mitragynine (1 and 30mg/kg) treatments. Among the proteins that were up-regulated in treatment groups were four calcium-binding proteins, namely calretinin, F-actin, annexin A3 and beta-centractin.

**Conclusions:** Upregulation of calretinin acted as low Ca^2+^ buffering upon the blockage of Ca^2+^ ion channel by mitragynine in the brain, which subsequently caused a reduction of GABA released and inversely increased the dopamine secretions that contributed to dependence indicators.

## Introduction

Kratom (*Mitragyna speciosa*) is one of Southeast Asia native herbs. Traditionally, rural residents consumed kratom leaves for its recreational properties. It was also used for treating common illnesses such as fever, cough, diarrhea, and discomfort 1,2. Given its putative pain-relieving and mood-altering properties, kratom is increasingly used in the United States (US) to treat chronic pain, opioid use disorder (OUD) and withdrawal, as well as anxiety and depression 3,4. Multiple studies have shown that persistent kratom intake can lead to dependence 4,5. FDA reported that among kratom withdrawal symptoms are irritability, aggression, tears, runny nose, difficulty working, jerky motions of the limbs, and aches in the muscles and bones 6,7. Fatalities due to kratom as the sole intoxicant are uncommon although hundreds of deaths related to kratom that involved more than one drug usage was reported by the Center for Disease Control and Prevention (CDC) of the United States 7,8. Nevertheless, despite the FDA's repeated calls to criminalize kratom under the Control Substances Act, there is no proof that kratom use has caused significant health problems such as those of classical opioids 9.

Kratom leaves contain approximately 40 types of alkaloids 10. Mitragynine is the most abundant indole alkaloid 11 consisting of 66% of the alkaloids content 11. 7-Hydroxymitragynine (7-HMG), an active metabolite of mitragynine, is made up 0.04% of the alkaloid content of kratom leaves extracts 11-13. 7-HMG is formed as the break down product of mitragynine by the activity of CYP3A4 14. In addition, 7-HMG may be formed post-harvest in kratom products that possibly contributing to increased reports of kratom toxicity in the Western world 14. Mitragynine has 9 times lower binding affinity to mu receptors (MOR) compared to 7-HMG, mitragynine is an MOR antagonist while 7-HMG is a partial agonist 15. The analgesic effect of mitragynine is mediated partially via mu and delta opioid receptors 16. Other physiological effects of mitragynine include ileum motility inhibition 3, smooth muscle contracture 17, and gastric acid secretion inhibition 18.

Cellular opioid dependence resulting from continuous stimulation of opioid-regulated signaling networks may ultimately lead to alterations in protein activities 19,20. It has been shown that repeated exposure to an addictive substance has modified the quantity or type of proteins expressed in specific brain areas 21. Such change in protein expression controls the activities of individual neurons that linked the brain circuits, from which may result in behavioral abnormalities associated with addiction 22. Amongst which are calcium-binding proteins that were expressed in many brain regions, and in various neuronal types in rodent 23. Thus, identifying the proteins related to the development and maintenance of drug dependence is critical not only in understanding the molecular mechanisms underlying addiction but also for developing of new pharmacological means to reverse the addictive state, prevent relapse, or reduce the use of these drugs 24.

We aimed to study the behavior of rats upon treatment with mitragynine, the main alkaloid constituent of kratom. Subsequently, the brain protein profile of the animals will be compared to the untreated rats to identify the differential expression of proteins. Such protein(s) is hoped to provide information on the possible mechanism of dependence or addiction on mitragynine, or both, which have not been documented.

## Materials and methods

### Animals

Animal study was carried out in compliance with the Institutional Animal Care and Use Committee rules and regulations [Reference number: USM/Animal Ethics Approval/2020/ (125) (1091)]. The rats were purchased from Universiti Sains Malaysia's Animal Research and Service Centre (ARASC) in Penang, Malaysia. A total of 48 male Sprague-Dawley rats (weighing 200 to 300g) were tested. They were naive and only utilized in one experiment. Under typical laboratory circumstances, they were socially housed in groups of 4 per cage in a temperature-controlled environment (24 ± 1 C). The room was kept on a standard 12 h light/12 h dark cycle. Before the experiments, the animals were handled for a week. Food and drink were freely available.

### Drug preparation

Mitragynine was extracted, separated, and validated from fresh *Mitragyna speciosa* leaves at the Centre for Drug Research, University Sains Malaysia, Penang, Malaysia. High-performance liquid chromatography (HPLC) and proton nuclear magnetic resonance (1H-NMR) (400 MHz) examinations showed 98 percent mitragynine purity 10,25. Mitragynine was dissolved in Tween 80 (20% v/v) as a vehicle. The doses of mitragynine (1 and 30 mg/kg) were given intraperitoneally.

## Experimental design

### Experiment I: Groups design

Pilot research was undertaken to determine the mitragynine behavioral implications 26. Daily injections of mitragynine (1, 30 mg/kg) and morphine (10 mg/kg) were given for 1 and 4 days in separate groups of rats. Selection of treatment durations were based on previous studies by Effendy et al. (2021) and Yusoff et al. (2014) 26,27. Tween 80 (20%) was injected into the negative control group. The behavioral signs were assessed and recorded for 30 minutes after 1 hour of mitragynine, morphine, and Tween 80 (20%) treatments.

#### Assessment of motor activities and indicators of dependence and addiction

One hour after the last treatment doses, the recording began. For 30 minutes, rats were placed in an open field test box (Automated open field, Pan Lab, USA), and withdrawal behavior was assessed. Chewing, head shakes, exploring, digging, yawning, teeth chattering, wet dog shakes, writhing, and test indicators such as squeaking on touch, hostility on handling, and diarrhea were included in behavioral scoring to determine physical dependency. Counted and checked signs were evaluated using the previously described scoring technique. The findings were multiplied by the relevant weighting factors to determine the intensity of addictive behaviors 10,28,29.

#### Behavioral analysis

The data were presented as mean ± standard error of the mean (SEM). Paired Student's t-test was used for open field test and Two-way ANOVA was used for counter and sign checks. In addition, One-way ANOVA was used to assess the global behavior scores. A significance level of p < 0.05 was used to test for statistical significance. GraphPad Prism 9.0 software (GraphPad Software Inc., La Jolla, CA, USA) was used to perform the statistics.

### Experiment II: Proteomic analysis of brain samples from treatment groups

#### Rats' brain collection

All rats treated with mitragynine, and control groups were sacrificed with pentobarbital (60 mg/kg, i.p.) an hour after the final treatment 26. The brains were collected as soon as possible and cleaned in normal saline before being shocked frozen in liquid nitrogen. The fatty layer around the brain was carefully removed to avoid undesirable influence during protein analysis. The tissues were stored at -80°C for further examination.

#### Brain Protein extraction

Three hundred microliters of Thiourea Lysis Buffer (TLB) [8 mM urea, 2 mM thiourea, 4 % (w/v) 3- [(3- cholamidopropyl) dimethylammonio] pH 3-10, 50mM, -1-propanesulfonate hydrate (CHAPS), 0.4 percent (w/v) carrier ampholytes 1,4-dithiothreitol (DTT)] was added at a ratio of 1:1.5 to 0.2 g of brain powder; tissue (weight): buffer (volume). Using a hand plastic homogenizer, the mixture was homogenized in an icebox, the mixture was centrifuged for 35 minutes at 14,000 rpm. The supernatant was collected and stored at -80 °C. One hundred mL of brain protein extract was added with 0.8 mL cold acetone containing 20 mM dithiothreitol (DTT). Trichloroacetic acid (TCA) in 100 mL was added to the mixture and thoroughly stirred. The mixture was then incubated for 100 minutes at -20°C. After incubation, the mixture was centrifuged for 15 minutes at 4°C at 14,000 rpm. The pellet was recovered, washed three times in cold acetone with 0.5 mL of 20 mM DTT. It was then centrifuged at 14,000 rpm for 15 minutes at 4°C. The pellet was air-dried for 30 minutes. The pellet was then reconstituted in 150 mL of TLB buffer. The mixture was incubated for 2 hours at 4°C and thoroughly mixed by brief vertexing for 20 min. The mixture was then centrifuged at 13,000 rpm for 15 minutes at 4°C. After centrifugation, the supernatant was collected in a microcentrifuge tube and subjected to protein concentration determination using an RC-DC protein assay (Bio-Rad, USA).

#### Two-dimensional polyacrylamide gel electrophoresis (2D-PAGE)

Six hundred µg of the protein extract in 125 µL TLB buffer containing a trace amount of Bromophenol Blue was passively rehydrated unto a 7 cm 4-7 immobilized pH gradient (IPG) strip (Bio-Rad, USA) for 15 hours. The IPG strip was then transferred to PROTEAN i12 IEF Cell (Bio-Rad, USA) for the focusing process 30. The focusing protocol followed 31 with slight modification as follow: 150V rapid climb for 1 h, 200V with a linear climb for 1 h, 500V with a linear climb for 1 h, 4000V with a linear climb for 2 h, 4000V with a linear climb until 10,000V was reached and then hold at 500V. After focusing, the IPG strip was treated with equilibration buffer I (6 M urea, 0.375 M Tris pH 8.8, 2% SDS, 20% glycerol, and 2% DTT) and subsequently with equilibration II (6 M urea, 0.375 M Tris pH 8.8, 2% SDS, 20% glycerol, and 2.5% iodoacetamide). The second-dimension separation was carried out on 12% SDS-PAGE using a constant voltage of 160 V throughout the run. The gel was stained using Coomassie Blue solution (0.01% Coomassie brilliant blue R250, 45% (v/v) methanol and 10% (v/v) glacial acetic acid) for 30 min at room temperature and distained with 50% (v/v) methanol and 2% (v/v) acetic acid for until the gel background turned colorless.

#### In-gel digestion

In-gel digestion of the target protein spots was performed according to the method of 32 . A clean blade was used to remove the spots from the gel. The excised gel fragments were rinsed for 10 minutes with 100 mM ammonium bicarbonate (NH_4_HCO_3_). After the buffer was discarded, the gel fragments were dehydrated using acetonitrile (ACN), then discarded after 5 minutes. The gel fragments were vacuum dried in a centrifuge (Eppendorf, Germany). The dehydration and washing processes were performed four times. Finally, the gel fragments were vacuum centrifuged to dry completely (Eppendorf, Germany). The dried gel fragments were soaked in 30 µl of 100 mM ammonium bicarbonate containing 10 mM DTT for an hour in a water bath at 56 °C. After removing the supernatant, the gel fragments were incubated for 45 minutes at room temperature in the dark in 30 µl of 100 mM ammonium bicarbonate containing 55 mM iodoacetamide. The previous steps of hydration and dehydration were repeated twice. Then, in digestion buffer (50 mM NH_4_HCO_3_, 5 mM CaCl_2_), 15 ng/µL trypsin was added to the gel pieces and incubated on ice for 1 hour. After discarding the trypsin solution, a volume of digestion buffer without trypsin was added to cover the gel pieces and incubated overnight at 37°C. The next day, the supernatant was collected into a clean vial, and peptides were extracted from the gel fragments using four cycles of 5% (v/v) formic acid in a 7:3 ACN: dH2O solution. Each cycle involved incubating gel fragments for 20 minutes before spinning them down and collecting the supernatant. All supernatants were recovered and dried at 37°C under a constant nitrogen gas flow.

#### LC/MS/MS analysis

The dried material was reconstituted with 30 µl of 0.1 % formic acid in HPLC grade water prior to LC/MS/MS analysis. The LC/MS/MS analysis was carried out using a Finnigan LTQ LC/MS/MS Easy-nLC II (Thermo Scientific, USA) system, with minor modifications to the method reported by Choi et al., 2015 33. Easy column C18 (10cm, 0.75mm i.d., 3µm; Thermo Scientific, San Jose, CA, USA) was conditioned at a flow rate of 0.3µl/min for 4 µl, whereas Easy column C18 (2cm 0.1mm i.d., 5µm; Thermo Scientific, San Jose, CA, USA) was used as the pre-column, it was conditioned at a flow rate of 3 µl/min for 15µl. The running buffers A and B were 0.1% FA in deionized water and 0.1% FA in acetonitrile, respectively. Fifteen µl of samples were injected into the columns and separated in 30 minutes at a flow rate of 0.3 µl/min with a gradient of 5 % to 100 % solvent B. With a capillary temperature of 220 °C and a source voltage of 2.1 kV, the eluent was interphase to the mass spectrometer. Peptides were identified using data-dependent LC/MS/MS with full scan mass analysis from m/z 300-2000 at a resolving power of 60,000. (ITMS). Charge states that were single or unassigned were discarded. Collision-induced dissociation was utilized as the fragmentation approach, with a collision energy of 35. Database matching was carried out using PEAKS studio Version 7 (Bioinformatics Solution, Waterloo, Canada). Peptide matching was performed using a database acquired from Uniport. Fixed alterations were Carbamidomethylation and oxidation. The maximum number of miss-cleavages was set to two, and the maximum number of variables after translation medication was set to three.

### Western blot

Protein extraction was conducted with 1 mL of lysis buffer [0.5 M Tris-HCl (pH 7.5), 50 μL of 1 M magnesium chloride (MgCl_2_), 10 μL of 1 M dithiothreitol (DTT), 100 μL of 0.2 M sodium orthovanadate (Na_3_OV_4_), 100 µL of Triton-X 100, 8.4 mL of deionized water, and 400μL of protease inhibitor cocktail]. The brain tissue was then homogenized on ice at a 1 g of brain tissue ratio per 9 mL of ice-cold lysis buffer 34 until the tissue powder was thoroughly lysed. The samples were centrifuged for 10 minutes at 8000 g at 4°C. The supernatant was transferred to a new tube. The protein content was determined using RCDC method (Bio-Rad). The supernatants were split into small aliquots and kept at -80°C deep freezer until analysis. SDS-PAGE was used to separate proteins from 30 μg of protein samples loaded onto a polyacrylamide gel using Laemmli method 35. The gel was soaked in cold transfer buffer [25 mM Tris, 192 mM Glycine, and 20% (v/v) methanol] for 30 minutes while nitrocellulose membrane (0.45μm) and blotting papers of 8.0 x 7.4 cm in size (Bio-Rad, USA) were soaked in transfer buffer for 30 min. Wet blotting was used to transfer proteins from gel to nitrocellulose membrane at 90 volts for 45 minutes. The membrane was then rinsed with washing buffer Tris-buffered saline/ Tween 20 (TBST) and gently agitated for 1 hour at room temperature with blocking buffer [5 % Blocker casein (skim milk) in TBST]. After blocking, the membrane was rinsed with washing buffer (TBST) three times. The membrane was then soaked in 1:1000 dilution of TBST rabbit anti-calretinin (Invitrogen, Thermo Fisher Scientific - US) at 4°C overnight with gentle agitation. After being washed three times with washing buffer, the membrane was socked in 1:2000 dilution of TBST goat anti-rabbit antibody (Invitrogen, Thermo Fisher Scientific - US) for 90 minutes at room temperature with gentle agitation. Finally, the membrane was treated with Opti-4CN substrate (Bio-Rad, USA) for 15 minutes with gentle shaking until the protein band was visualized.

### Statistical Analysis

#### Image analysis

ChemiDoc^TM^ Imaging System (Bio-Rad, USA) was used to capture all the 2D gel images. The images were analyzed using PDQuest Software version 7.3. (Bio-Rad, USA). 2D-gel images of protein isolated from the controls and treatment groups were used to create match sets. The standard and differentially expressed protein spots were compared and identified between the negative control and treatment groups. All of the gel images were normalized to minimized background interference. Every protein spot's intensity was calculated as a percentage of the overall intensity of valid spots. Differential protein expression was evaluated using protein spot intensity analyzed using One-way ANOVA at p<0.05 significance level.

## Results

### Experiment 1: Assessment of motor activity and addictive behaviors

**Figure 1:**
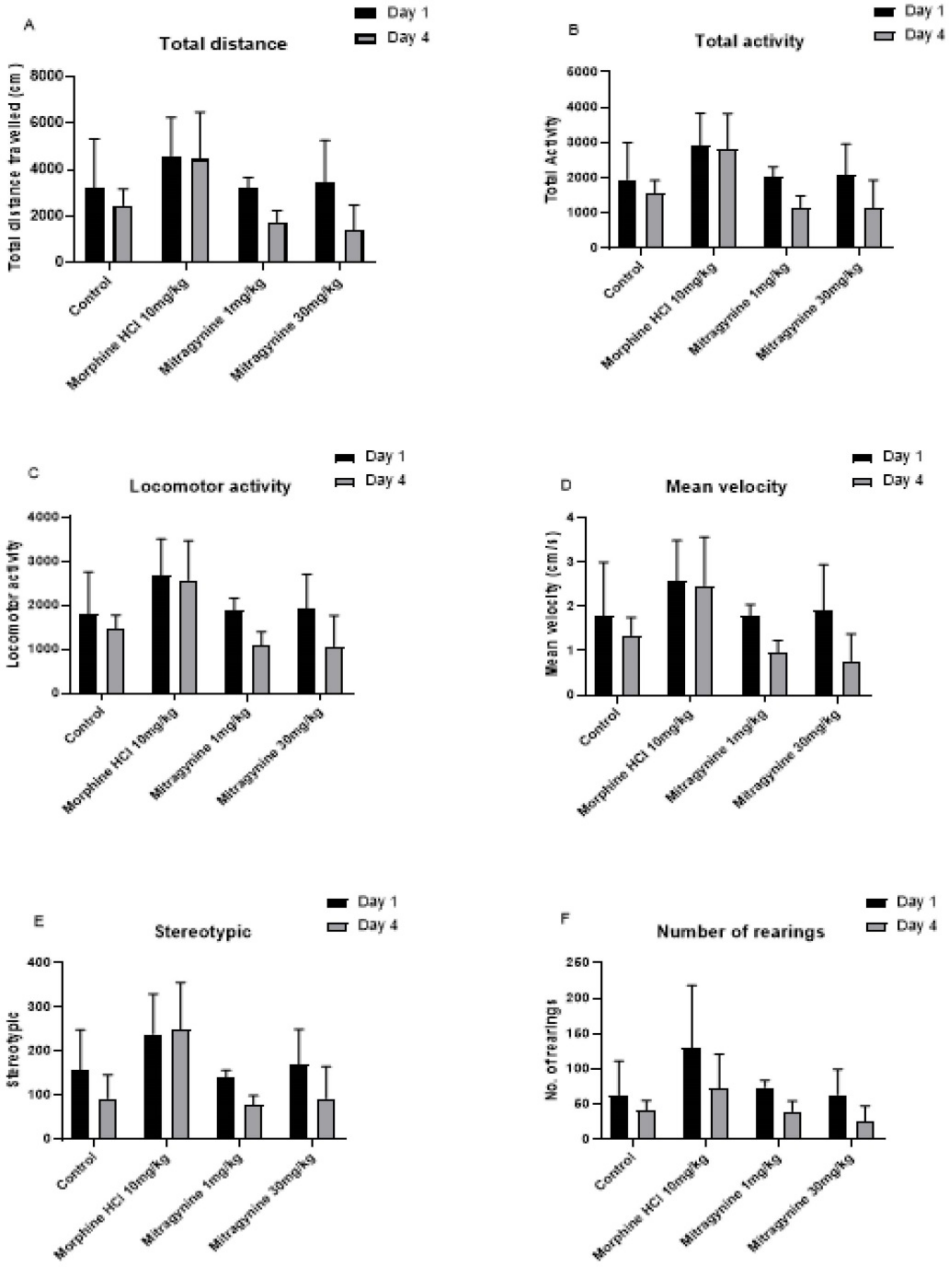
Effects of vehicle, morphine (10mg/kg) and mitragynine (1 and 30mg/kg) treatments on motor activities using automated open-field task on A) total distance travel, B) total activity, C) locomotor activity, D) mean velocity, E) stereotypic and F) number of rearing. Data are expressed as mean ± SEM from rats (n = 6-8) per group and analyzed using the unpaired Student t-test.

The open-field test was performed one hour following treatments with the vehicle, mitragynine (1 and 30mg/kg), and morphine (10mg/kg) on days 1 and 4 for a period of 30 minutes. Spontaneous movement and activity of the animal were measured after drugs consumption. Tasks such as total distance travelled, total activity, locomotor activity, mean velocity, stereotypic (repetitive) movements, and rearings were recorded (Fig 1A to F). Paired Student's t-test showed no significant differences in total distance travelled (p = 0.125, Fig. 1A), total activity (p = 0.125, Fig. 1B), locomotor activity (p = 0.125, Fig. 1C), mean velocity (p = 0.125, Fig. 1D), stereotypic behavior (p = 0.25, Fig. 1E), and number of rearing (p = 0.125, Fig. 1F).

Two-way ANOVA revealed significant behavioral score for head shakes in the group treated with morphine 10 mg/kg for 4 days (p = 0.0495); writhing in the groups treated with mitragynine 1 mg/kg and 30 mg/kg for 4 days (p = 0.0495), and morphine 10 mg/kg for 4 days (p = 0.0495). The results of the one-way ANOVA revealed a significant treatment between groups with F 6, 21 = 2.796; p = 0.0369 for the global withdrawal scores (Table 1).

### Experiment 2: Proteomic analysis of brain samples

This study analyzed the proteome of proteins extracted from brain tissues that were separated by 2D gel electrophoresis. The brain proteomes of rats treated with mitragynine, and morphine (positive control) were compared to those of negative control rats. The upregulated calcium-binding proteins from the brain proteome of the low dose (1 mg/kg) and the high dose (30 mg/kg) of mitragynine treated rats were circled in red (Fig. 3). Compared to the negative control rats, these protein spots were found significantly upregulated and consistently presented with > 2 folds intensity in all the animals. These protein spots were excised from the gel and subjected to in-gel digestion with trypsin enzyme, and LC-MS/MS analysis was performed on the digested protein fragments. Table 2 shows the identity of the four protein spots, the proteins were calretinin, F-actin, annexin A3 and beta-centractin. For 1 day and 4 days treatment groups, the expression of these four proteins in the low dose (1 mg/kg) and high dose (30 mg/kg) mitragynine rats was consistently presented with significantly upregulation intensity (p<0.05) in all the rats. Fig. 4 shows an example of the MS and MS/MS spectrum, respectively, for calretinin.

Table 3 shows the protein expression levels of the four calcium-binding proteins according to their spot intensity. Each gel was loaded with an equal quantity of protein (600 µg). Analysis on the intensity of the protein spots showed that at 1 day treatment with mitragynine at low and high doses, significant changes in the intensity of all the four calcium binding protein spots (p<0.05) was observed when compared to the negative control group. Similarly, for 4 days treatment with mitragynine, both the low and high doses caused significant up-regulation (p<0.05) in the calcium binding proteins' expression levels as indicated by spot intensities of the four proteins when compared to the negative control group. When comparing between day 1 and day 4 treatments, the expression of these proteins was found significantly higher in day 4 treatments groups for both low and high doses of mitragynine when compared to their corresponding treatments groups in day 1. When comparing within the day 1 treatments groups, there was no significant difference in the intensity of the proteins in low and high doses of mitragynine. On the contrary, the 4 days treatments groups showed a significant upregulation (p<0.05) of all the four calcium-binding proteins at high dose of mitragynine compared to low dose mitragynine. The Western blot experiment confirmed the identity of calretinin. The calretinin antibody was found binding to the calretinin band at 29kDa (Fig. 5A). The binding of the calretinin antibody to the calretinin band, as shown by the intensity, indicated that a higher expression of calretinin was detected in the treatment groups. Fig.5B shows the bar chart of calretinin expression in negative control, and high dose of mitragynine for 1 day and 4 days treatment. The data showed that at 30 mg/kg high dose of mitragynine, the expression of calretinin was significantly higher (p<0.05) in 4 days treatment compared with 1 day treatment and the negative control group.

## Discussion

Mitragynine made up 66% of the total alkaloids content in kratom 11. Although mitragynine binds to opioid receptors, this indole alkaloid is structurally and pharmacodynamically different from its opioid rival. Therefore it is identified as atypical opioid that distinguishes it from morphine, semisynthetic opioids, and endogenous ligands 36. Upon binding to opioid receptors, the indole alkaloid activates G-protein-coupled receptors (GPCRs). Nevertheless, unlike conventional opioids, indole alkaloids do not initiate the β-arrestin pathway when they activate GPCRs 37. β-arrestin recruitment is responsible for the symptoms of opioid use, such as respiratory depression, sleepiness, and constipation 38,39. Kratom, like opioids or some stimulants, may cause dependency 40. In view of the high quantity of indole alkaloid content of kratom, kratom addiction mechanism pathway may not be identical to those of conventional opioids. Nevertheless, the use of single alkaloid of kratom poses limitation to this study. The data presented here may not fully illustrate the effects of kratom on animal behavior or brain proteome changes caused by kratom consumption, although the study of singular alkaloid (mitragynine) would have the advantages of isolating the effects of this single alkaloid.

**Figure 2:**
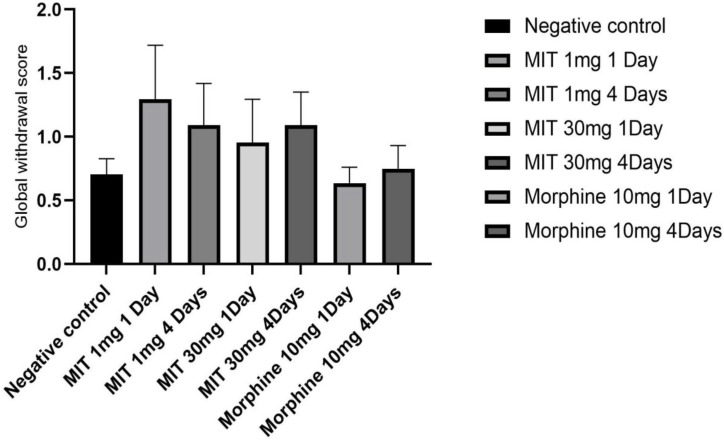
Single behavior ratings were generated and translated into a global withdrawal score to reduce variability and increase the reliability of withdrawal signs outcomes. Data are expressed as mean ± SEM from rats (n = 6-8) per group and analyzed using one-way ANOVA.

**Figure 3:**
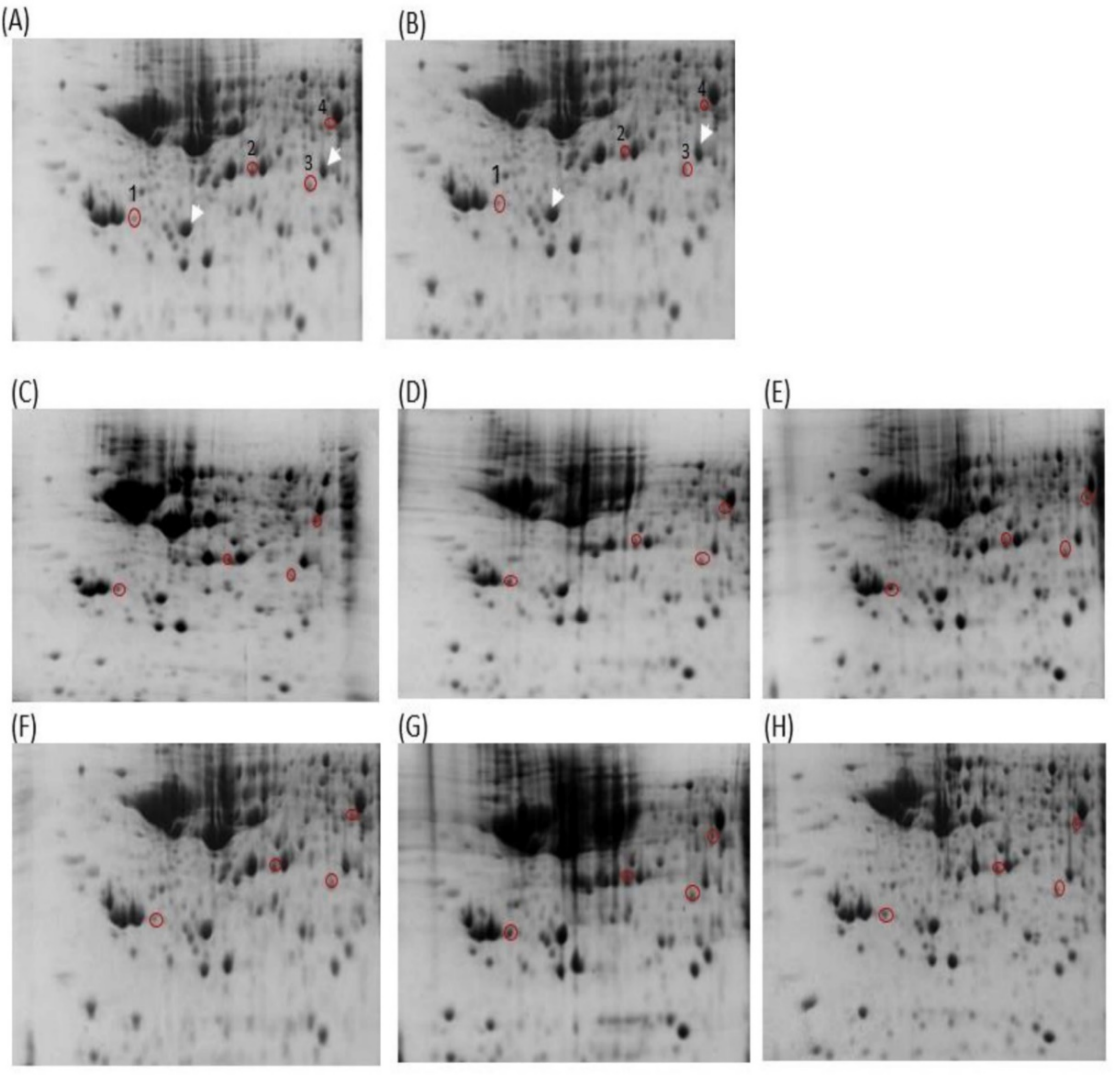
2D gel images of Thiourea Lysis Buffer (TLB) extracts: (A) negative control 1 day, (B) negative control 4 days, (C) mitragynine (MIT) 1 day (1mg/kg), (D) MIT 1 day (30 mg/kg), (E) morphine 1 day (10 mg/kg), (F) MIT 4 days (1mg/kg), (G) MIT 4 days (30 mg/kg), (H) morphine 4 days (10 mg/kg). The white arrow denoted the landmark used in gel image analysis. Red circles denoted the up-regulated proteins. The spot number was labeled accordingly to the protein listed in Table 2.

**Figure 4:**
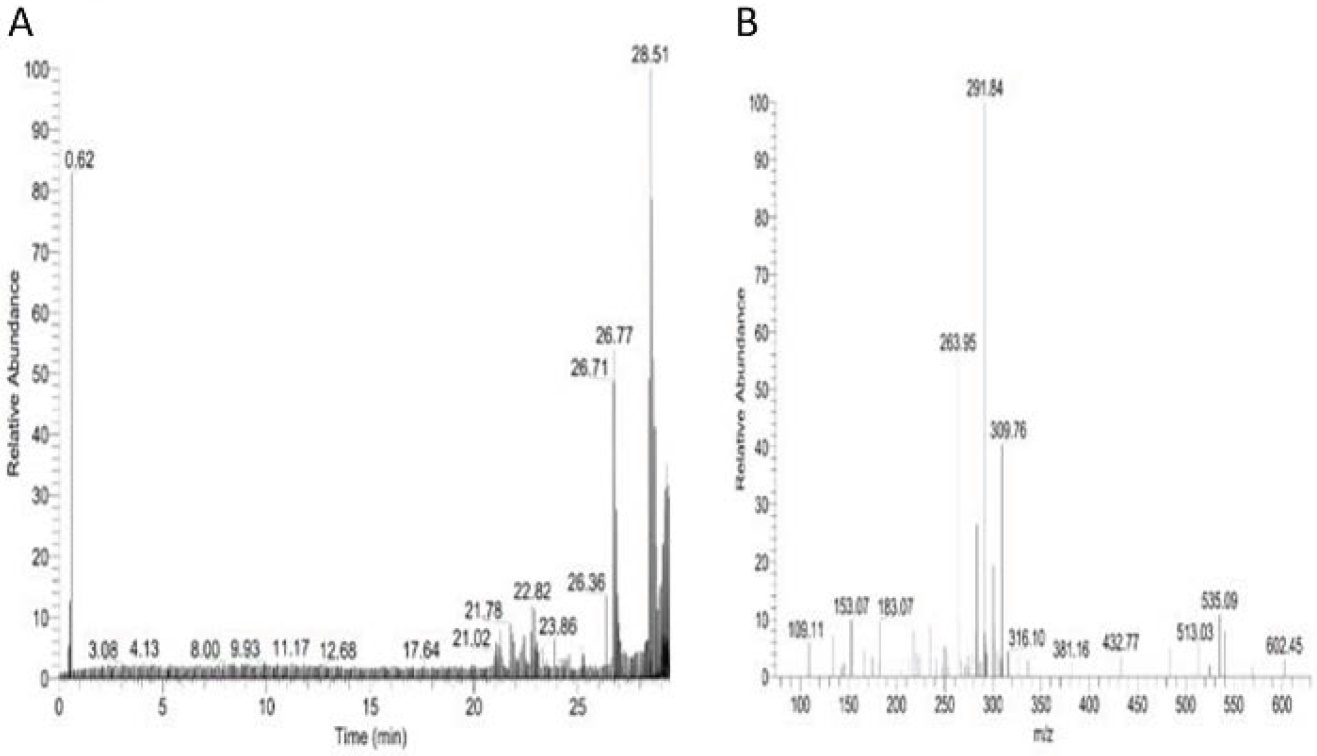
LC/MS/MS analysis for brain calretinin. Panel A. shows the total ion chromatogram of brain calretinin; Panel B. shows an MS/MS spectrum of one of the peptides of brain calretinin.

**Figure 5:**
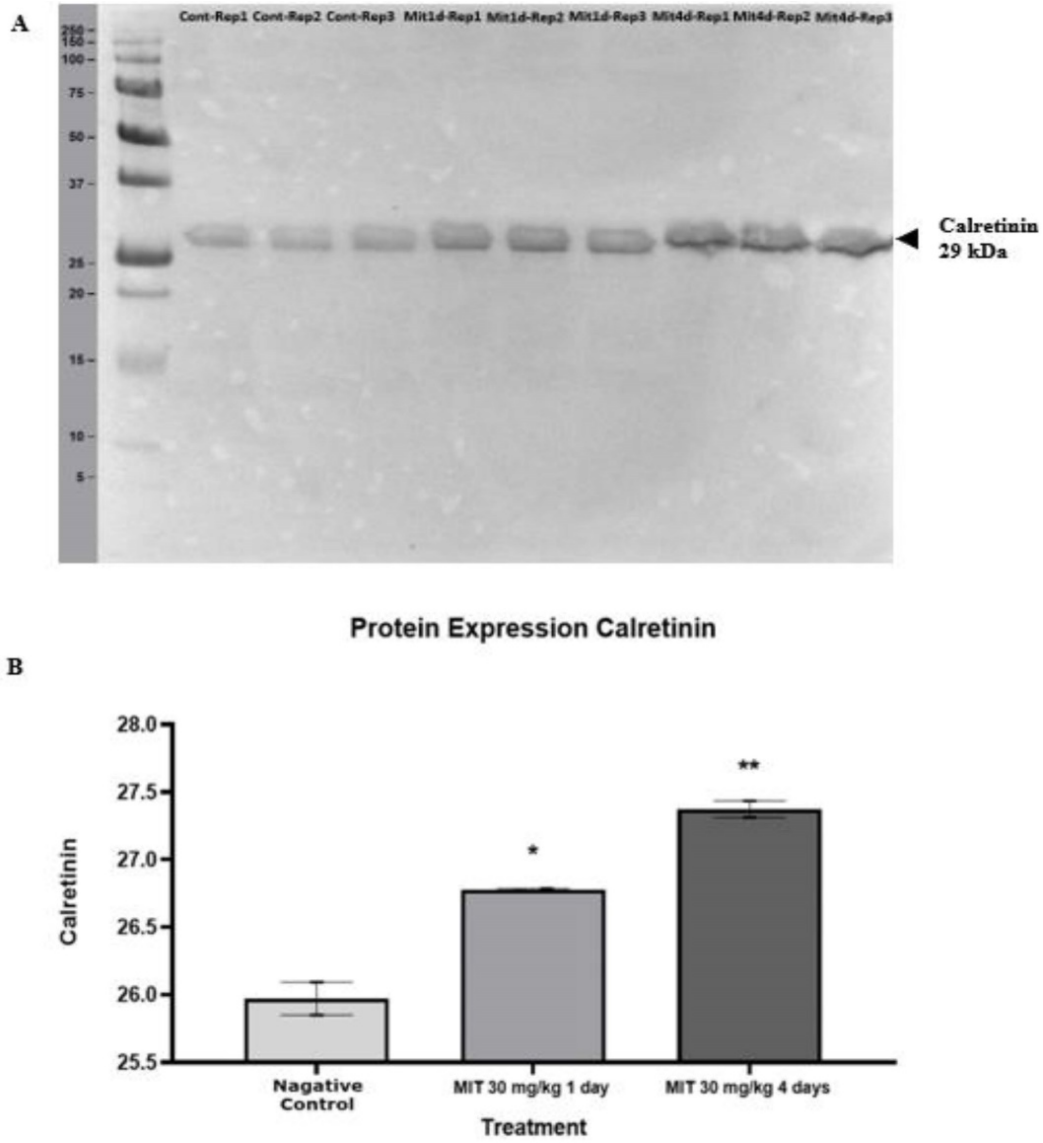
(A) The Western blot analysis of the calretinin at 29 kDa extracted from rats' brain tissues. (B) A statistical representation of Western blot analysis of the protein expression. Calretinin expression in mitragynine (30 mg/kg) treated groups (1^st^ day * p < 0.05, 4^th^ day ** p < 0.01) was significantly higher compared to negative control group.

In this study, the behavioral responses of rats to single and multiple doses of mitragynine at low (1mg/kg) and high (30mg/kg) doses were evaluated and subsequently the differential brain proteins expressions of the rats were analysed. The rats' behavior was recorded and scored day 1 after a single dose (both high and low doses) or day 4 following repeated administration of mitragynine (both high and low doses). Morphine was used as the positive control substance to study the behavioral changes of the animal upon treatment with addictive substance. There were no significant differences in motor activity of rats between single and repeated mitragynine treatment by using open field tasks. Nevertheless, both rats in low and high mitragynine doses showed slightly reduce motor activity on day 4. The severity of withdrawal after a single and repeated doses of mitragynine was evaluated to assess animal physical dependence. The behavioral scoring data revealed that the rats developed physical dependence only on day 4 following morphine and mitragynine (1 and 30mg/kg) treatments. This is seen when the number of writhing decreases dramatically compared to the negative control. The morphine and mitragynine treatments groups reduced withdrawal discomfort following a single (high dose) and repeated (both doses) therapy by lowering the number of writhes. In order to reduce variability and to improve dependability of the results, single behavior scores were calculated and translated into a global withdrawal score (Figure 2), where no significant difference was found between the groups (p>0.05).

In the second part of the study, the brains of the rats were harvested, and their brain proteome were analyzed using 2D gel electrophoresis. The brain proteomes of the morphine and the treatments groups at low dose (1 mg/kg) and high dose (30 mg/kg) of mitragynine for day 1 and day 4 revealed that a few protein spots were expressed at higher intensity compared to the negative control group. The protein spots were excised from the gel and subjected to mass spectrometry analysis. Among these proteins, four belonged to calcium-binding proteins. All these protein spots were consistently present at higher intensities in the mitragynine and morphine groups when compared to the negative control group at significant levels (p<0.05). These calcium-binding proteins were calretinin, F-actin, annexin A3 and beta-centractin. When comparing between days of treatment, the 4 days treatment group showed a significantly (p<0.05) higher expression of the proteins for both low and high doses of mitragynine than the day 1 treatment groups. When comparing between low and high doses mitragynine treatments on the same day, the high dose mitragynine treatment for day 4 showed a significant (p<0.05) upregulation of all the four proteins compared to the low dose mitragynine. In contrary, on 1 day treatment groups, there was no significant difference in protein expression in both doses of mitragynine treatments.

Our previous study by Hassan et al., (2019) 10 stated the importance of Ca^2+^ influx in the initiation of the most forms of long-term potentiation (LTP) and short-term potentiation, which included changes in the postsynaptic calcium levels, such as those seen during LTP induction. Another study by Matsumoto et al. (2005) 41 reported that mitragynine blocks T- and L-type Ca^2+^ channel currents and reduces KCl-induced Ca^2+^ influx in neuroblastoma cells. Based on these findings, changes in Ca^2+^ signaling showed to be responsible for the impairments in hippocampal transmission and plasticity and memory acquisition observed with mitragynine administration. Hence, Ca^2+^ plays a significant pharmacological role in mitragynine treatment rats. Consequently, the up-regulation of the brain's calcium binding proteins that were reported in this study may be the result of the lowering of Ca^2+^ concentration due to the blocking of the Ca^2+^ ion channel upon ingestion of mitragynine. Both annexins 42 and actin 43 are involved in Ca^2+^ channels regulation, while centractin is characterized as actin-related protein 44. All these proteins were detected up-regulated in our current study.

**Figure 6:**
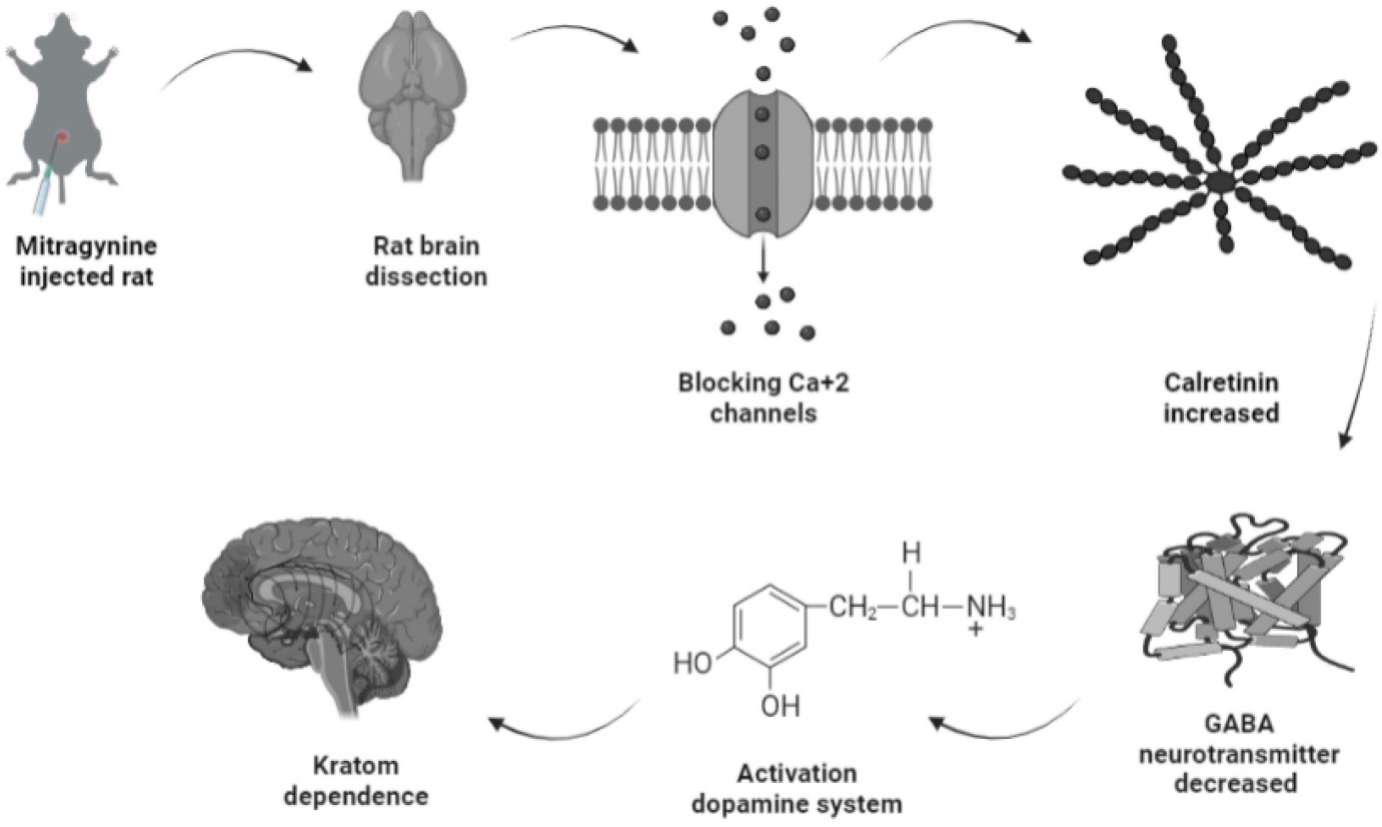
Possible pathway of kratom dependence.

Amongst the four upregulated calcium-binding proteins, calretinin is expressed on the excitability neurons 45. Structurally, calretinin is a calcium-binding protein which contains 6 EF-hand domains, four of them bind to Ca^2+^ with high affinity, one is not binding to Ca^2+^, while one was non-functional and without Ca^2+^ binding affinity 46,47. With such a characteristic, calretinin shares some kinetic properties of both slow and fast buffers in modifying dendritic Ca^2+^ transients 48. Upon binding of Ca^2+^ to calretinin, the mammalian neuronal cytoplasmic concentration of calretinin was estimated to be in order of tens of micromoles 49 and it was shown to be highly concentrated beneath the cell membrane 50, which affects intracellular calcium signals both pre- and postsynaptically 51-55. One putative biological function of calretinin was its role in the modulation of neuronal excitability. Modulation of calcium signaling by calretinin has been reported to play a critical role for precise timing and plasticity of synaptic events in neuronal networks 45. A study by Schurmans et al., (1997) 51 has reported that LTP induction of mice lacking calretinin (calretinin -/- null mutant mice) was impaired following tetanic stimulation of hippocampal inputs. LTP of excitatory synaptic responses of principal neurons in the hippocampus is accompanied by changes in GABAergic inhibition mediated by interneurons 56. Therefore, upregulation of calretinin in the mitragynine treated rats as reported by this current study will concomitantly strengthen LTP induction and thus results in reduction of GABA neurotransmitter being released. This will inversely induce the secretion of dopamine neurotransmitter unto granule cells. While activation of dopamine system was reported to play a role in several behavioral including depression, ADHD, and various forms of addiction 57, which may explain the dependence of rats to mitragynine as displayed by their behaviors. The demonstration of this possible pathway of mitragynine dependence is described in Fig. 6.

Systems that regulate intracellular Ca^2+^ levels are part of the complex Ca^2+^ signaling network. They include gated Ca^2+^ channels, energy-dependent pumps, and intracellular Ca^2+^ binding proteins that act as controlled Ca^2+^ buffers. Amongst these, Ca^2+^ binding proteins are more directly involved in Ca^2+^ signaling because their characteristics change in response to Ca^2+^ binding 58. Furthermore, neuroprotective effect of calretinin against cellular damage mediated by very low Ca^2+^ concentration has been described 59,60. The upregulation of calretinin may indicate its neuroprotective effect towards the cellular damage in the condition of inhibition of Ca^2+^ influx to the cellular system by treatment with mitragynine.

It was showed in this study that the opioid effects of mitragynine as referenced to the positive control substance, morphine, causing behavioral changed in the rats only at high dose for 1 day treatment or 4 days continuous administration of mitragynine at high and low doses. These concentrations of mitragynine may be needed to cause an upregulation of calretinin to a threshold that manifested as drug dependence behaviors similar to those of morphine.

## Conclusion

The upregulation of calcium-binding proteins may be the direct response of the brain to the lack of Ca^2+^ in the brain upon mitragynine ingestion by the treatment rats. The upregulation of calretinin may potentially explain the cellular mechanism to regular or high dose mitragynine ingestion which correlated to the dependence behavior of the rats. This possible mechanism of mitragynine dependence behavior will be further investigated in our future study.

## Figures and Tables

**Tab1e 1:** Results of the counted and checked signs for evaluation of the severity of withdrawal signs for negative control, mitragynine (MIT) (1 and 30mg/kg) and morphine (Mor)(10mg/kg) for days 1 and 4. Data are expressed as mean ± SEM from rats (n = 6-8) per group and analyzed using Two-way ANOVA.

Days	Day 1				Day 4			
Behavioral activity	Negative Control	MIT 1mg/kg	MIT 30mg/kg	Mor 10mg/kg	Negative Control	MIT 1mg/kg	MIT 30mg/kg	Mor 10mg/kg
Chewing	2 ± 0.00	1 ± 057	3.5 ± 0.95	2 ± 0.00	2 ± 0.00	1.5 ± 0.5	4 ± 0.81	3 ± 0.57
Head shakes	4± 0.816	5 ± 1.29	4 ± 1.41	2.5 ± 0.5	4± 0.820	4.5 ± 0.95	5.5 ± 0.95	1 ± 0.57 *
Exploring	1 ± 0.00	1 ± 0.00	1 ± 0.00	1 ± 0.00	1 ± 0.00	1 ± 0.00	1 ± 0.00	1 ± 0.00
Digging	0.00 ± 0.00	0.00 ± 0.00	0.00 ± 0.00	0.00 ± 0.00	0.00 ± 0.00	0.00 ± 0.00	0.00 ± 0.00	0.00 ± 0.00
Yawning	0.00 ± 0.00	0.00 ± 0.00	0.00 ± 0.00	0.00 ± 0.00	0.00 ± 0.00	0.00 ± 0.00	0.00 ± 0.00	0.00 ± 0.00
Teeth chattering	0.5± 0.5	0.5 ± 0.5	0.00 ± 0.00	0.00 ± 0.00	0.5± 0.5	0.5 ± 0.5	0.00 ± 0.00	0.00 ± 0.00
Wet dog shakes	0.00 ± 0.00	0.5 ± 0.5	0.5 ± 0.5	0.00 ± 0.00	0.00 ± 0.00	0.5 ± 0.5	0.00 ± 0.00	0.00 ± 0.00
Writhing	6 ± 0.81	5.5 ±1.25	1 ± 0.5*	1± 0.57*	6 ± 0.82	3± 0.57 *	1± 0.57*	3.57 ± 0.57 *
Squeaking on touch	0.00 ± 0.00	0.25± 0.25	0.00 ± 0.00	0.00 ± 0.00	0.00 ± 0.00	0.5 ± 0.28	0.00 ± 0.00	0.00 ± 0.00
Hostility on handling	0.5 ± 0.288	0.5 ± 0.28	0.5 ± 0.28	0.5 ± 0.28	0.5 ± 0.283	0.5± 0.28	0.25 ± 0.25	0.25 ± 0.25
Diarrhea	0.00 ± 0.00	0.00 ± 0.0	0.00 ± 0.00	0.00 ± 0.00	0.00 ± 0.00	0.00 ± 0.00	0.25 ± 0.25	0.00 ± 0.00

**Table 2:** List of up-regulated calcium-binding proteins detected in TLB extract.

NO.	Protein ID	Protein name	Accession	Protein function
1	299787	Calretinin	P47728|CALB2	the modulator of neuronal excitability including the induction of long-term potentiation.
2	308407	F-actin	Q3T1K5|CAZA	cell stability and morphogenesis.
3	300852	Annexin A3	P14669|ANXA3	differentiation, cell migration, immune regulation, and bone formation
4	309415	Beta-centractin	Q8R5C5|ACTY	cellular functions

**Table 3:** The protein expression levels of the four calcium-binding proteins according to their spot intensity.

Groups		Calretinin	F-actin	Annexin A3	Beta-centractin
1 day	Negative Control	1641±0.56	1297±0.35	1537±0.7	1354±0.29
	Morphine 10 mg/kg	4243±1.00^***^	4803±0.017^***^	4491±0.61^***^	4211±0.82^***^
	Mitragynine 1 mg/kg	2490±1^***^	2515±0.76^***^	2781±0.45^***^	2291±0.93^**^
	Mitragynine 30 mg/kg	2835±0.95^***^	3020±1.00^***^	2385±0.87^**^	3031±0.76^***^
4 days	Negative Control	1599±0.90	1312±0.55	1579±0.90	1299±0.77
	Morphine 10 mg/kg	5490±0.59^***^	6926±0.91^***^	5216±1.50^***^	4089±2.00^***^
	Mitragynine 1 mg/kg	3477±0.63^***^	3536±0.34^**^	2551±0.55^*^	2471±0.89^***^
	Mitragynine 30 mg/kg	4145±0.57^***^	3369±0.33^**^	2792±1.00^**^	2938±0.70^***^
